# Ultrasound-Assisted Extraction of Anthocyanins Using Natural Deep Eutectic Solvents and Their Incorporation in Edible Films

**DOI:** 10.3390/molecules26040984

**Published:** 2021-02-12

**Authors:** Patricia Velásquez, Daniela Bustos, Gloria Montenegro, Ady Giordano

**Affiliations:** 1 Departamento de Química Inorgánica, Facultad de Química y de Farmacia, Pontificia Universidad Católica de Chile, Santiago 7820436, Chile; pdvelasquez@uc.cl (P.V.); dcbustos@uc.cl (D.B.); 2 Departamento de Ciencias Vegetales, Facultad de Agronomía e Ingeniería Forestal, Pontificia Universidad Católica de Chile, Santiago 7820436, Chile; gmonten@uc.cl

**Keywords:** carrageenan, berries, green chemistry, green extraction, active compounds, medicinal plants, active packaging

## Abstract

Extracts rich in bioactive compounds added to edible films have allowed the development of active packaging that increases the shelf life of food. However, it is necessary to search for solvents that are nontoxic and not harmful to the environment, with natural deep eutectic solvents (NADES) being an attractive and easily synthesized alternative. This research aimed to design NADES by lyophilization to be used in the extraction of anthocyanins from the Chilean *Luma chequen* (Molina) A. Gray berry, and subsequently adding them to the matrix of edible ƙ-carrageenan films. For this purpose, ultrasound-assisted extraction (UAE) was used and the anthocyanin content was evaluated with the pH differential method. The antioxidant capacity of extracts was determined by DPPH assay and the antibacterial capacity by diffusion agar tests. The results obtained indicate that the designed NADES are efficient at extracting anthocyanins, reaching concentrations between 81.1 and 327.6 mg eq cyanidin 3-glucoside/100 g dw of *L. chequen* (Molina) A. Gray. The extracts reached inhibition diameters between 5 and 34 mm against *Escherichia coli*, *Staphylococcus aureus*, and *Salmonella typhi* strains. Once the extracts were incorporated into ƙ-carrageenan films, active edible films with antioxidant and antibacterial capacities were obtained.

## 1. Introduction

An active edible film is defined as a sheet or thin membrane that is wrapped on the surface of food [1] to extend its shelf life, reducing the processes of oxidation, antimicrobial spoilage, gas exchange, and moisture loss [2], in addition to improving sensory characteristics such as color, gloss, roughness, and mechanical properties, among others [3]. The impetus to create such packaging originated from environmental concerns caused by the use of plastics, in addition to consumer demand for healthier and more natural foods [2,3]. In this sense, edible films are based on biodegradable compounds that are safe for human consumption, such as polysaccharides, proteins, lipids, or mixtures of these [4,5], together with the addition of other compounds, such as plasticizers; the latter are used in order to modify the functional properties of the films and increase their extensibility, elasticity, and flexibility, as well as other mechanical properties [6,7]. The casting method is mostly used for its simplicity and low cost, and requires solvents for the solution and dispersion of the biopolymer on a flat surface, followed by a drying process under controlled conditions for removal of the solvent and formation of the film [5]. The recent incorporation of natural extracts of plants, fruits, and spices represents a promising approach for the development of active edible films, due to their high quantities of polyphenols, such as flavonoids, phenolic acids, and anthocyanins, which can improve the functionality of the film based on the antioxidant and antibacterial capacity of these compounds [6,7]. For this reason, there is currently a need to extract large amounts of bioactive compounds from plant resources using non-harmful and environmentally friendly solvents such as natural deep eutectic solvents (NADES) [8,9].

NADES are mixtures of primary metabolites such as sugars, organic acids, alcohols, amino acids, and amines, united by strong intermolecular interactions, particularly hydrogen bonds, together with other interactions such as van der Waals and electrostatic forces. There are various methods reported in the literature for the synthesis of NADES. The lyophilization method is based on freeze-drying the solution until a viscous liquid is obtained [10,11]. This method is often preferred for the extraction of heat-sensitive compounds such as anthocyanins [12,13]. The principal advantages of NADES are that their interaction confer excellent physical and chemical properties, such as low volatility, nonflammability, and adjustable viscosity [10,14,15], giving NADES solubilizing behavior that is different from other conventional solvents and has been used more recently in combination with ultrasound energy in order to increase extraction yield of natural products [16,17,18,19,20]. In particular, it has been seen that the use of NADES composed of combinations of quaternary ammonium salt-alcohol, quaternary ammonium salt-carboxylic acid and carboxylic acid-sugar have been used to extract anthocyanins from plant matrices. The most widely used, both in binary and ternary combinations, are citric acid, mal acid, lactic acid, glucose, fructose, xylose, glycerol and choline chloride [13,19,21,22,23]. An important point is that in most of the jobs UAE is used as an extraction method because it has been proven that it has the advantage of being green, fast and clean, which improves the yield of anthocyanin extraction [13,19,22,23,24,25,26].

Berries are often the richest source of antioxidants among fruits and vegetables, where Chile has several native species, such as chequén (*L. chequen* (Molina) A. Gray), calafate, maqui, and murta, that have been collected since pre-Columbian times as sources of food [27]. Chilean native fruits are naturally rich in phenolic compounds and have a high antioxidant power, as indicated by an investigation carried out by Simirgiotis et al. [28]. The high anthocyanin content would explain the traditional use of native species, mainly in the form of infusions that provide different benefits for human health. Anthocyanins (from the Greek *anthos*, flower, and *kianos*, blue) are the main water-soluble pigments responsible for the blue, pink, red, and violet color in the fruits and flowers of many plant species [29,30]; moreover, they are associated with the protection against biotic and abiotic stress [29]. However, one of the most important properties of anthocyanins is their antioxidant activity, which plays a vital role in the prevention of neuronal and cardiovascular diseases, diabetes, and cancer, among others [30]. Anthocyanin-rich edible films have proven to be suitable for decreasing food spoilage, since they have higher antioxidant activity than nonactive films [31].

The objective of this study was to extract anthocyanins from a Chilean berry with the use of specially designed NADES assisted with ultrasound, and to evaluate the antioxidant and antibacterial characteristics in edible films incorporating them. For this, 10 NADES were designed for the extraction of anthocyanins, selecting the composition by means of bibliographic exploration and synthetizing them through the lyophilization method. The anthocyanins were extracted, identified, and quantified by different methodologies. The antioxidant and antibacterial activities of the extracts were determined, and the combinations with the best properties were selected and added as part of the edible film matrix, evaluating the film’s bioactive properties.

## 2. Results and Discussion

### 2.1. NADES Performance in Anthocyanin Extraction

The results of the bibliographic search showed that the compounds most used in the preparation of NADES for anthocyanin extraction are those with high polarity and hydrophilicity, such as sugars, organic acids, and choline salts (See Section 3.3). A set of 10 NADES were designed, including lactic acid (La), glycerol (Gly), tartaric acid (Ta), glucose (Glu), choline chloride (CIC), and citric acid (Ca) in different ratios (Table S1). These solvents were selected because they have proven capacities as NADES to extract anthocyanins. Special consideration has been given to the compatibility of edible films in those cases where only solvents with an alimentary grade had been selected. Glycerol was included because it is an effective plasticizer used for polysaccharide-derived films [32,33]. All designed NADES exhibited different water contents, since water contributes to strengthening the network of hydrogen bonds in the system [34] with an increase in viscosity. However, a high percentage of water causes the breakdown of hydrogen bonds in the eutectic mixture, which leads to a supramolecular breakdown in the structure of solvents, weakening them [35].

When extracting the bioactive compound from the fruit *L. chequen* (Molina) A. Gray with the NADES set, it was observed that the anthocyanin content varied with the relationship between solvent components (Table 1); the extracts with the highest anthocyanin extraction capability corresponded to La:Glu 8:1, ClC-Gly 4:6, Gly:Glu 8:1, and Ta-Gly 1:4. Most of these solvents have in common a high proportion of glycerol and water in their matrix; previous works reported that NADES based on polyalcohols showed improved extraction of bioactive compounds [36]. Glycerol is often used in the synthesis of NADES as a cheap and ecological alternative [37]. In addition, it has applications in different fields such as cosmetics, pharmaceuticals, and the food industry, where it is used as a humectant, thickener, and sweetener, among others [37]. It should be noted that extracts prepared in glycerol can be incorporated into food or cosmetics without the need for an extra procedure to eliminate residual solvent [38].

Glucose, choline chloride, and tartaric and lactic acids also appear in NADES compounds with the best performance in extracting anthocyanins from *L. chequen* (Molina) A. Gray, probably related to the previously described contributions to the solvent’s properties. Glucose is widely used in the production of NADES, as it is a good electron donor. Organic acids contribute to the formation of a greater number of intermolecular interactions and to the stability of liquids formed due to the presence of hydroxyl and carboxylic groups in their structure [39].

In a study by Bosiljkov et al. (2017) [22], the use of NADES was evaluated for the extraction of anthocyanins from residues of the wine industry, which indicated that NADES based on organic acids were the best solvents in this case. The importance of an acidic environment for the extraction and stability of anthocyanins [22] was address by Panić et al. [23] during the development of an extraction method for anthocyanins from grape pomace using eight NADES based on different organic acids. In both studies, it was observed that NADES based on organic acids could fully address the isolation and stability requirements of anthocyanins, since under these conditions (pH < 2), the flavyl cation form prevails [22,23], while when the pH rises between 3 and 4, the structures around the flavyl cation change to form the quinoidal species [22,30].

The pH value of the NADES used in Panić’s research [23] was between 0.49 and 3.27, very similar to synthesized NADES, which have a pH ranging from 0.27 to 4.45. Regarding the total anthocyanin content, the concentration range varied between 0.28 and 0.92 mg/g in grape pomace [23]. For *L. chequen* (Molina) A. Gray, the total anthocyanin concentration range varied between 2.26 and 3.30 mg/g with the use of NADES based on tartaric and lactic acids. More importantly, this value is higher than the 1.16 mg/g extracted when using a traditional solvent as ethanol (Table 1), and the one previously reported by Simirgiotis et al. [28]. More recently, when methanol was used as a solvent, the total anthocyanin content obtained was 1.54 mg g^−1^ for myrtle berry [27]. When evaluating the chromatographic profiles (Supplemental Materials), four characteristic signals can be observed for the ethanolic extract of *L. chequen* and for the extracts with NADES that could correspond to cyanidins, delphinidins and petunidins previously reported by Ramírez et al. [40] and Simirgiotis et al. [28]. Moreover, one or two additional initial signals are observed with respect to the chromatographic profile of the ethanolic extract of *L. chequen*, which would indicate the presence of a greater number of anthocyanin compounds extracted by the methodology used, indicating the difference in the extraction capacity between the ethanol and NADES used.

According to these results, the extracts with the highest anthocyanin content (mg eq cyanidin 3-glucoside/100 g dw of fruit) were selected for the next steps, since they differed significantly from the rest: La-Gly 1:2, Ta-Gly 1:4, La-Glu 8:1, Gly-Glu 8:1, ClC-Ca 5:4, and ClC-Gly 4:6.

### 2.2. Color and Anthocyanins

The polymeric color (Equation (3)) is a value that expresses the color of an anthocyanin when treated with a bleaching agent such as sodium bisulfite [41]. On the other hand, the color density (Equation (4)) expresses the color imparted by a monomeric and polymerized anthocyanin, and is obtained from the sum of absorbances at λ vis-max and λ 420 nm obtained when treating the sample with distilled water [41]. Table 2 lists the values obtained for each extract.

It can be seen that the polymeric color for extracts La-Glu 8:1 and Gly-Glu 8:1 is higher than the rest, which indicates that the anthocyanins are much more resistant to the presence of nucleophilic species such as sodium bisulfite; therefore, part of the imparted color is associated with the presence of polymeric anthocyanins. 

Regarding the color density, the values obtained are in ranges of 1.91 ± 1.18 and 3.65 ± 1.46, indicating than the color observed in the extract is due to the action of monomeric and polymeric anthocyanins and other reaction products. Therefore, it is directly related to the contribution of the polymeric color. Extracts with less polymeric color are the ones that have lower color density values, so it can be inferred that these extracts have greater amounts of monomeric anthocyanins providing color and not so many polymerized ones.

With these values, the percentage of polymeric color was obtained, since it is an indication of the degradation of anthocyanins [41]. The percentage in the extracts varied in a range between 25.49 and 55.22%. It should be noted that the extracts Gly-Glu 8:1 and ClC-Gly 4:6 had a much higher percentage of polymeric color compared to the rest of the extracts (*p* < 0.05), which is related to the pH of these combinations (pH > 4; Table S1). In conditions closer to pH 7, the cation form is the predominant and most stable species, contributing to the red-purple coloration of anthocyanins; however, it must be considered that the higher the percentage of polymeric color, the higher the degree of polymerization and degradation.

Given the importance of the relationship between the stability and color of anthocyanins in the extracts, the parameters L*, a*, b*, *H°*, and C* were determined (Table 2). The parameter L*, or luminosity, is an indication of light and dark color [42]. The values varied between 18.85 ± 0.34 and 27.48 ± 0.47. Most of the extracts presented similar values, with the exception of the extracts ClC-Gly 4:6 and Gly-Glu 8:1, which presented much higher values and are precisely those extracts that appear to have less coloration to the naked eye. For this parameter, a positive association with pH was observed, as indicated by the Pearson correlation (r = 0.67; data not shown); that is, as we increase the pH, the luminosity increases. This behavior is mainly evidenced in these extracts, which were made with a pH greater than 4.

Regarding the coordinate a*, its positive values go toward reddish tones and negative values toward green tones. Most of the extracts have high values due to the stabilization of the flavillus cation at acid pH and with a predominance of red color [43], with the exception of the extracts ClC-Gly 4:6 and Gly-Glu 8:1, with a* coordinate values of 7.07 and 8.88, respectively. The rest of the extracts did not have significant differences with respect to this parameter. Most of the extracts had similar b*, chromaticity values, and hue angles, with no significant differences.

### 2.3. Antibacterial and Antioxidant Activity of Anthocyanin Extracts

**Figure 1:**
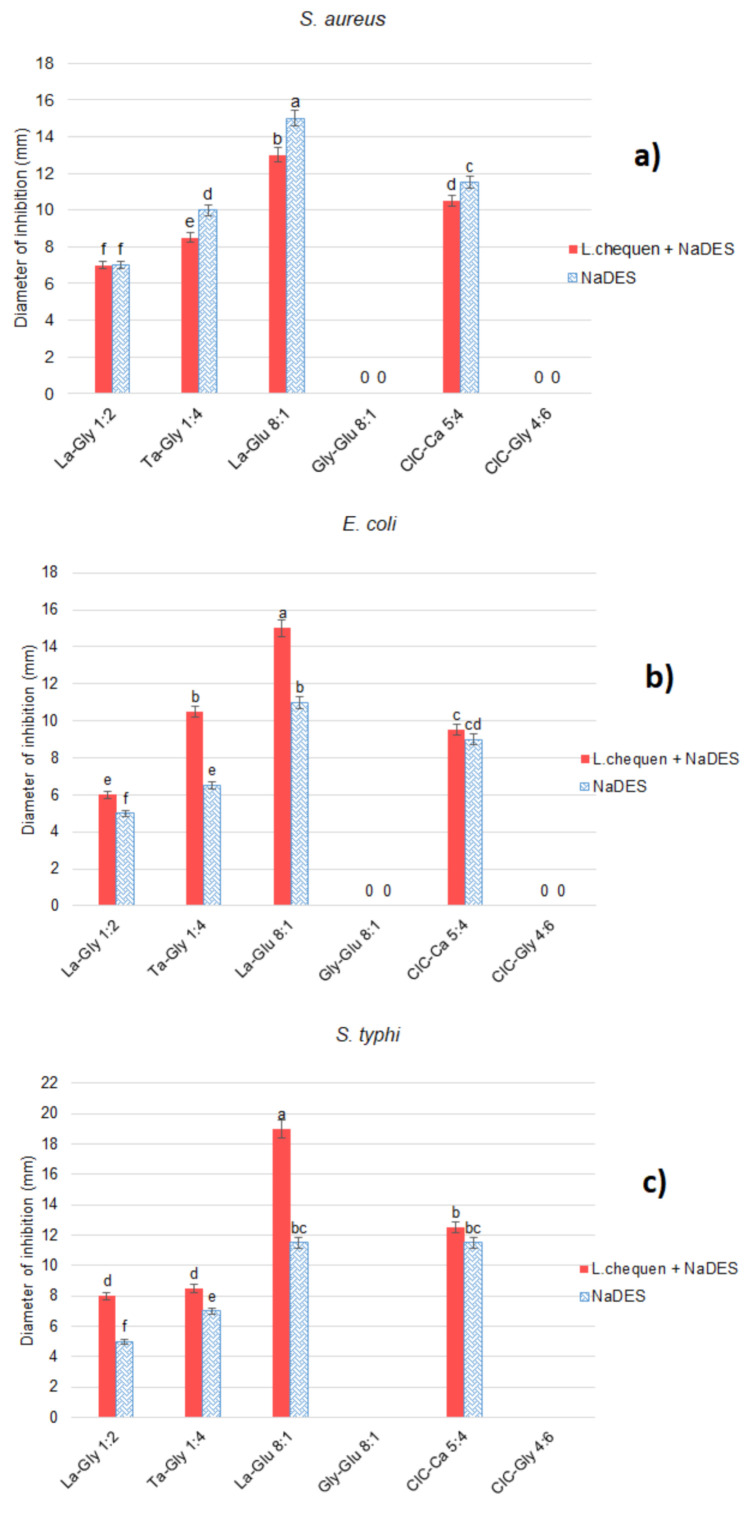
Antibacterial capacity of extracts of *L. chequen* obtained with NADES listed in Table 2 against (**a**) *S. aureus*, (**b**) *E. coli*, and (**c**) *S. typhi* (different letters in columns indicate significant difference (Tukey’s HSD, *p* < 0.05)).

Antibacterial capacity was measured by the inhibition halos (mm) of the extracts of *L. chequen* (Molina) A. Gray against three bacterial strains: *E. coli, S. typhi*, and *S. aureus*. As can be seen in Figure 1, most synthetized NADES showed activity against all three bacteria. For the *E. coli* strain, it was observed that as the concentration of anthocyanins extracted from *L. chequen* increased in a range from 60.11 to 82.66 mg/L, the inhibition halos increased from 1 to 8 mm, respectively, differing significantly (*p* < 0.05) in the halos obtained by the NADES. Regarding *S. typhi*, the inhibition halo varied from 2 to 17 mm with respect to the NADES. This increase shows a direct dependence on the concentration of total anthocyanins in each extract, thus demonstrating a significant effect, since increased halos are observed due to the action of the bioactive compounds in the extract as well as the solvent.

For the three bacteria, the highest inhibition was caused by La-Glu 8:1 extract, composed mainly of lactic acid and glucose, indicating an evident role of the composition of the solvent used in the extraction. The antibacterial activity of organic acids has been reported, with the undissociated fraction of the acid showing the highest activity due to its lipophilic nature, since it can cross the cell membrane and dissociate in the cytoplasm of the microorganism [44]. On the other hand, the high osmolarity of glucose also makes it an optimal component for the inhibition of such bacteria [45].

Gly-Glu 8:1 and ClC-Gly 4:6 extracts did not show antibacterial activity. In both cases, the lack of activity was due to the high pH value in relation to the other extracts. This is in accordance with the negative association found between pH and antibacterial activity, as indicated by Pearson’s correlation (r = −0.79); that is, as pH increases, antibacterial activity decreases, showing a statistically significant (*p* < 0.05) dependence. Lacombe et al. [46] reported that the inhibitory action of berries, such as blueberries, against microorganisms is linked to acidic pH. In this sense, the extracts that were made with these two NADES did not show antibacterial activity, since the anthocyanins suffered structural changes and instability, making it impossible to achieve destabilization of the cytoplasmic membranes of the microorganisms or inhibit their growth [47]. All synthesized NADES and extracts showed higher activity than the ethanolic extract, which in the same experimental conditions showed a diameter of inhibition of 1 mm for all bacteria assayed.

The minimum inhibitory concentration (MIC) was determined for extracts with activity in the inhibitory halo evaluation (Table S3). Regarding the MIC value of the extracts obtained (*L. chequen* + NADES), for *E. coli* and *S. typhi* strains, similar values were determined when using La-Gly 1:2 or Ta-Gly 1:4, with MIC values of 0.878 and 0.439 μg extract/mL, respectively. For the *S. aureus* strain, MIC values between 1.755 and 0.439 μg extract/mL were determined. For the rest of the extracts, it was not possible to determine the MIC values, in agreement with what was observed in the agar diffusion tests (Figure 1): larger inhibition halos indicate better antibacterial power. It should be noted that there are no reports indicating the antibacterial power of the *L. chequen* (Molina) A. Gray fruit. Therefore, the results obtained in this study are crucial to add new information regarding the potential for the use of this type of berry. In the same experimental conditions, ethanolic solvent extract showed MIC of 1.56 mg/mL for *S. aureus* and 3.12 mg/mL for *E. coli* and *S. typhi*. These results are significantly higher than the values obtained for NADES extracts, approximately one order of magnitude less effective against the bacteria.

Inhibition curves of the DPPH radical were constructed with different concentrations (data not shown), from which the IC_50_ index (Table S3) was determined, which represents the amount of extract necessary to capture the DPPH radical by 50% [48,49]. The extracts showing the highest inhibition were La-Gly 1:2 and Ta-Gly 1:4, with 3.45 and 4.21 mg/mL, respectively. This is attributed to the presence of anthocyanins and other phenolic compounds in *L. chequen* (Molina) A. Gray extracts, as reported by studies [49,50] where Chilean berries were studied, such as maqui and murtilla, and identified as a rich source of beneficial phenolic compounds for human health, with the most prominent being phenolic acids, flavonoids, flavonols, and anthocyanins. This study only quantified the anthocyanins present in extracts; therefore, it is possible that other phenolic compounds affected the antioxidant activity of the analyzed extracts.

A study by Lillo et al. [51] used the DPPH radical method to evaluate the antioxidant capacity of native berries, including maqui (*Aristotelia chilensis*), myrtle (*Luma apiculata*), and murta (*Ugni molinae*), highlighting maqui with an IC_50_ value of 3.70 mg/mL, followed by murta with 6.97 mg/mL and *L. apiculata* with 8.08 mg/mL. The reported values are similar to those obtained for *L. chequen* fruits in the present report.

Additionally, it must be considered that in this study, the determination of antioxidant capacity was carried out on extracts prepared with different solvents, which had an influence on the values obtained for individual samples. This influence was due to the fact that single electron transfer (SET) and hydrogen atom transfer (HAT), key aspects of antioxidant capacity, are affected by the type and polarity of the solvent [52], which added to the efficiency of extraction of bioactive compounds.

### 2.4. Influence of L. chequen (Molina) A. Gray Extracts on Edible Films

For the preparation of edible films, the casting method was used, and its formulation included ƙ-carrageenan as a biopolymer for the matrix and a mixture of equal parts glycerol and polyethylene glycol as plasticizers. It should be noted that there are no studies incorporating bioactive extracts based on NADES as functional additives. The use of these solvents as plasticizers has only been reported in the synthesis of films based on chitosan and pectin [32,53].

The films made in this research presented different appearance and flexibility due to the composition of the added extract. In general, most of the films were easy to manipulate and detach from their mold; however, films made with a ClC-Ca 5:4 extract, based on choline chloride and citric acid, were very rigid and brittle and were, thus, discarded. 

The antioxidant activity of the edible films was studied by the percentage of inhibition of DPPH and ABTS radicals (Table 3). In the antioxidant capacity analysis using DPPH, no significant differences were observed between evaluated samples (*p* > 0.05); that is, all analyzed films had similar effectiveness in reacting with the DPPH radical, with inhibition percentages ranging between 7.26 and 10.81%, for a concentration of 6.25 mg/mL of control film-forming solution.

Considering the ABTS methodology, higher percentages of inhibition were observed, specifically for the film made with La-Gly 1:2 extract, at 30.50%, followed by the film with Gly-Glu 8:1, at 23.85%. This difference in inhibition is explained by the low selectivity of ABTS+, which reacts with any hydroxylated aromatic compound whether or not it has potential as an antioxidant, unlike DPPH [52]. Regarding the inhibition percentages obtained from the control film compared to films with extracts, there was an increasing trend that can be associated with the presence of functional groups with antioxidant properties, such as hydroxyl from the extract [31], as previously described. Li et al. [54] incorporated natural antioxidants into edible gelatin-based films, highlighting green tea extracts, mainly for their high polyphenolic compound content, and grapeseed extract, for its abundance of tannins and flavonoids, such as epicatechin and catechin, as being very effective in increasing the antioxidant activity of the films. Propolis extract and anthocyanins extracted from red cabbage were used as natural additives in films made with polyvinyl alcohol and starch [55], which improved their mechanical and barrier properties, and they presented good antibacterial activity against bacteria transmitted in food. Similarly, de Araujo et al. [56] used ethanolic extract of propolis in cassava starch films, showing that the antioxidant capacity of the films was proportional to the concentration of the extract, and that after the casting process, the propolis retained its antioxidant activity.

It should be considered that this study evaluated a new type of additive that included a matrix different from those reported; therefore, the inhibition percentages obtained are not comparable. Even so, possibilities open up for the use of these extracts, including the anthocyanins and NADES solvent used, since they contribute to improve the antioxidant capacity of the films.

The antibacterial activity of the edible films against the three bacterial strains increased when *L. chequen* extracts were added, as evaluated by the diffusion disk method. As shown in Table 3, the control *ƙ*-carrageenan film showed no inhibitory activity against the evaluated pathogens. This behavior is similar to that reported by Liu et al. [7] for a control film based on a ƙ-carrageenan matrix without the addition of pomegranate (*Punica granatum* L.) peel and pulp extracts.

The effect of incorporating extracts into the polymeric matrix of ƙ-carrageenan reflects the NADES used and the anthocyanins extracted. Films made with 3.5% extract showed zones of inhibition between 5 and 7 mm, and *E. coli* was the most sensitive to the films. Several investigations have shown the effect of adding bioactive compounds to edible films, such as a study by Kanmani and Rhim [57], who evaluated the antibacterial activity of edible films based on ƙ-carrageenan with the incorporation of seed extracts of grapes. and observed wide inhibition halos against Gram-positive bacteria such as *L. monocytogenes* and *S. aureus*. Similarly, Liu et al. [7] reported inhibition halos between 5.1 and 7.1 mm for Gram-positive and Gram-negative bacteria of edible films made with extracts of pomegranate peel and pulp associated with the presence of polyphenolic compounds.

It should be noted that the inhibition diameters described in Table 3 do not correspond to halos formed around the film disk but to the inhibition zone formed by the film disk in contact with the microorganism. This behavior has been shown in films based on chitosan, where the antibacterial effect occurs without active agents migrating through the agar; therefore, only microorganisms in direct contact with the active sites of the polymeric matrix are inhibited [58,59].

Better results were expected because when evaluating the antibacterial capacity of the extracts, broad inhibition halos were observed. It should be noted that the extract was incorporated into a polymeric matrix; therefore, it is possible that new interactions were formed that prevented correct diffusion in the agar, causing the action of the anthocyanin extract to be directly from the film. Most solvents used are glycerol-based NADES, which are also widely used as plasticizer. If the extract was evaluated as another plasticizer when introduced between the ƙ-carrageenan chains, the number of interactions increased due to the hydrogen bonds directly influencing the diffusion capacity. Therefore, to improve this analysis, it is necessary to further investigate the interaction between the components and the structural changes in the film when the extract is incorporated, along with an evaluation of its physical mechanical properties.

## 3. Materials and Methods

### 3.1. Chemical Reagents and Solvents

Glycerol (C_3_H_8_O_3_), lactic acid (C_3_H_6_O_3_), glucose (C_6_H_12_O_6_), tartaric acid (C_4_H_6_O_6_), potassium metabisulfite (K_2_S_2_O_5_), polyethylene glycol, 2,2′-azino-bis(3-ethylbenzothiazoline)-6-sulfonic acid (C_18_H_24_N_6_O_6_S_4_), pH 4.5 buffer (sodium acetate (C_2_H_3_NaO_2_)–glacial acetic acid (C_2_H_4_O_2_)), and ƙ-carrageenan were purchased from Sigma-Aldrich (St. Louis, MO, USA). Choline chloride–citric acid and choline chloride–glycerol were purchased from Ionchem Solvents (Santiago, Chile). A pH 1.0 buffer (38% HCl, hydrochloric acid), potassium chloride (KCl), HPLC grade methanol (CH_3_OH), ethanol, acetonitrile (C_2_H_3_N), formic acid (CH_2_O_2_), and Mueller-Hinton agar were supplied by Merck (Darmstadt, Germany).

### 3.2. Plant Material

Mature fruits of *L. chequen* (Molina) A. Gray were sustainably collected at the end of March 2019 in the V Region of Valparaíso, and identified by Professor M. Gómez, an expert on native Chilean flora. The samples were transferred to the natural products laboratory of the Faculty of Agronomy and Forest Engineering (Pontificia Universidad Católica de Chile, Santiago, Chile) and left in a conventional freezer for 24 h. They were then lyophilized (Labconco FreeZone 1 L, Kansas City, MO, USA) at 0.06 mBar and −40 °C. Once the fruits were lyophilized, they were ground and stored at room temperature in the dark.

### 3.3. NADES Synthesis

The lyophilization method was employed to NADES synthesis [60]. Each component selected by previously studies [11,14,17,19,20] was mixed in a certain molar ratio (Table S1) and stored at −80 °C for 2 h. Subsequently, they were lyophilized (−40 °C, 0.006 mbar) for 18–24 h until a homogeneous and viscous liquid was obtained. For pH determination, a pH meter (PL-700PC) was used.

### 3.4. Extraction of Anthocyanins by Ultrasound

For the extraction, 25 mg of powdered lyophilized fruit was weighed and 1 mL of each combination of solvents (NADES and ethanol) was added. The sample was sonicated (35 kHz; VWR Ultrasonic Cleaner (VWR international, Radnor, PA, USA)) for 60 min and subsequently centrifuged in a mini-centrifuge (40 W/10,000 rpm; Allsheng, Model 10K (Hangzhou Allsheng Instruments CO., Ltd., Hangzhou, Zhejiang, China)) for 30 min. The extract obtained was stored at −4 °C in the dark.

### 3.5. Chromatographic Profile of Anthocyanins by HPLC-DAD

For the identification of anthocyanins, the method proposed by Ramírez et al. [40] was followed, with modifications. Chromatographic profiles were obtained with a Hitachi LaChrom Elite high-efficiency chromatography device coupled to a diode array detector (model L-2455).

Ten microliters of each extract were injected into a LiChrospher C18^®^ column (250 mm × 5 mm × 4.6 mm). The chromatographic mobile phase was a mixture of 0.1% formic acid (A) and acetonitrile with 0.1% formic acid (B) at a flow of 1 mL/min, with the following gradient: 90% solvent (A) for 4 min, followed by 90–75% solvent (A) for 25 min, then 75–10% (A) for 35 min, and again 90% (A) for 45 min. Finally, the column was reconditioned with 90% solvent (A) for 15 min. Anthocyanins were monitored at 520 nm.

### 3.6. Total Monomeric Anthocyanins

The pH differential method is based on the structural changes manifested by anthocyanins in relation to pH variation [41].

For the quantification of total anthocyanins, two buffer solutions were prepared, one at pH = 1.0 and one at pH = 4.5. From each extract, 100 µL was taken and diluted to a volume of 300 µL with distilled water. Two 20 μL aliquots were taken and one was mixed with 280 μL of pH 1.0 buffer and one with 280 μL of pH 4.5 buffer in a 96-well plate. Each sample was allowed to equilibrate for 15 min, then the absorbance at 700 nm and λ vis-max was measured in a Cytation^TM^ 5 multimode reader (BioTek® Instruments, Inc., Winooski, VT, USA). The following Equations were used to calculate the concentration of total monomeric anthocyanins, expressed as cyanidin-3-glucoside equivalent:

(1) $$A=({\left(\;A_{\lambda \mathrm{vis}-máx}-A_{700}\;\right)}_{pH=1.0}-{\left(\;A{\;}_{\lambda \mathrm{vis}-máx}-A_{700}\;\right)}_{pH=4.5})\;$$

(2) $$Total\;anthocyanins\;\left(\frac{mg}{L}\right)=\frac{A\;\times \;MW\;\times \;DF\;\times \;1000}{\varepsilon \;\times \;1}$$

*MW* = 449.2 g/mol; *DF* = dilution factor, *ε* = 26,900 L/mol cm

### 3.7. Polymeric Color of Anthocyanins

A buffer solution pH = 1.0 and a 1 M sodium bisulfite solution were prepared [41]. From each extract, 100 µL was taken and diluted to a volume of 300 µL with distilled water. A 20 μL aliquot was mixed with 180 μL of pH 1.0 buffer and 100 μL of distilled water, and a second 20 μL aliquot was mixed with 180 μL of pH 1.0 buffer and 100 μL of sodium bisulfite. The samples were allowed to equilibrate for 15 min and then the absorbance at 420 nm, λ vis-max, and 700 nm was measured in the Cytation^TM^ 5 multimode reader. Polymeric color was calculated according to Equation (3), using data from the aliquot treated with bisulfite, while color density was calculated according to Equation (4), with data of the aliquot treated with water (DF = dilution factor).

(3) $$Polymeric\;color=\left[\left(A_{420}-A_{700}\right)+\left(A_{\lambda \;\mathrm{vis}-máx}-A_{700}\right)\times \;DF\right]$$

(4) $$Color\;density=\left[\left(A_{420}-A_{700}\right)+\left(A_{\lambda \;\mathrm{vis}-máx}-A_{700}\right)\times \;DF\right]$$

To determine the percentage of polymeric color, Equation (5) is used:

(5) $$\%\;Polymeric\;color=\frac{Polymeric\;color\;}{Color\;density}\;\times \;100$$

### 3.8. CIELab Color

The CIELAB coordinates (L*, a*, b*) of the NADES-based extracts were determined for the CIE illuminant D65 and 10° observer angle by using a tristimulus colorimeter (Portable FRU WR-10, Shenzhen Wave Optoelectronics Technology Co. Shenzhen, China), where L* represents lightness and varies between 0 (black) and 100 (white), a* expresses red (+) or green (−), and b* indicates yellow (+) or blue (−). Each extract was placed in a quartz cuvette for measurement. Chroma (*C°*) and hue (*H°*) values were obtained from the following Equations:

(6) $$C{}^{\circ}=\sqrt{a^{2}+b^{2}}$$

(7) $$H{}^{\circ}=arctan\frac{a^{*}}{b^{*}}$$

### 3.9. Antibacterial Capacity of Extracts

The antibacterial capacity was evaluated on the strains *Escherichia coli* ATCC-25922, *Staphylococcus aureus* ATCC-25923, and *Salmonella typhi* ATCC-700623.

#### 3.9.1. Agar Diffusion Method

To carry out the antibacterial study of the extracts, the agar culture medium (Müller-Hinton) was initially prepared and 25 mL was placed in 90 mm × 15 mm Petri dishes. Then, the inoculum of the strains was prepared in a saline solution until reaching similar turbidity for a concentration of 0.5 McFarland. With a sterile swab, it was sown in a parallel and compact way over the entire surface of the Petri dish and allowed to dry for about 3 min; previously, sterile Sensi-Discs of a diameter of 6 mm was impregnated with 10 μL of extract and had been placed on the agar. The plates were incubated at 37 °C for 24 h. All inhibition diameter were reported without including the 6-mm diameter of sterile paper discs or edible film.

#### 3.9.2. MIC

Dilution in broth was then carried out in order to determine the minimum inhibitory concentration (MIC) [46]. A 96-well microplate was used and a concentrated solution of 100 μL of broth, 100 μL of extract, and 50 μL of the inoculum of each strain was prepared, after which eight dilutions were prepared (1:2). The microplate was allowed to incubate at 37 °C for 24 h. Aliquots of 4.5 μL were taken from the dilutions and subcultured in Petri dishes previously prepared with 25 mL of agar, after which they were incubated at 37 °C for 24 h.

### 3.10. Antioxidant Capacity

Antioxidant capacity was conducted by decolorization method [48]. A 150 µM methanolic solution of DPPH was prepared. An aliquot of 200 μL was taken and mixed with 20 μL of each extract at different concentrations. After 30 min of incubation, the absorbance at 517 nm was measured in the Cytation^TM^ 5 multimode reader. Inhibition of the DPPH radical was evaluated by the following Equation:

(8) $$\%DPPH\;inhibition=\left(\frac{\;A_{0}\;-A_{1}}{A_{0}}\right)\;\times \;100$$

where *A*_0_ is the DPPH absorbance and *A*_1_ is the extract absorbance. With the results obtained, the IC_50_ value was determined by graphing the percentage of inhibition of DPPH vs. concentration of extract; by means of linear regression, the slope (*m*) and intercept (*b*) were obtained:

(9) $$IC_{50}=\left(\frac{50-b}{m}\right)$$

### 3.11. Edible Films

For the elaboration of edible films, the method reported by Fu et al. [61] was followed, with modifications. A 1% *w*/*v* solution of ƙ-carrageenan was prepared with constant heating and stirring at 80 °C for 60 min with the addition of 30% of plasticizer (50% glycerol + 50% polyethylene glycol) in relation to the dry mass of the biopolymer. Subsequently, the solution was cooled to 60 °C, and a 10 mL aliquot was taken and mixed with 350 μL of the extract (25 mg/mL dry weight), homogenized in a vortex, and poured into Petri dishes (90 cm × 15 cm) to be dried in an oven at 30 °C for 24 h.

### 3.12. Antioxidant Capacity of Edible Films

The antioxidant capacity of the films was evaluated by the DPPH and ABTS radical decolorization methods [48]. To evaluate the antioxidant power, samples were prepared by adding 4 mL of distilled water to 25 mg of edible film. Samples were homogenized in vortex and supernatants were taken for analysis.

#### 3.12.1. ABTS^+^ Radical Cation Method

A stock solution of ABTS was prepared, which consists of the reaction of an aqueous solution of ABTS (7 mM) with one of potassium persulfate (2.45 mM); 10 mL of each was taken and allowed to react for 12–16 h in the dark. One milliliter of the stock solution was taken and diluted with methanol. Then, 150 μL of the freshly prepared ABTS solution was mixed with 50 μL of the supernatant and allowed to react for 30 min, after which the absorbance was read at 732 nm with the Cytation^TM^ 5 multimode reader. This procedure was carried out in duplicate.

With the absorbance values, the percentage of inhibition was calculated using the following Equation:

(10) $$\%ABTS\;inhibition=\left(\frac{\;A_{0}\;-A_{1}}{A_{0}}\right)\;\times \;100$$

where *A*_0_ is the absorbance of ABTS and *A*_1_ is the absorbance of the sample.

#### 3.12.2. DPPH Radical Method

A methanolic solution of DPPH (150 µM) was prepared. Then, 20 μL of edible film supernatant was mixed with 200 μL of the DPPH solution and incubated for 30 min and the absorbance at 517 nm was read in a Cytation^TM^ 5 multimode reader. With the data obtained, the percentage of inhibition of the DPPH radical was calculated using Equation (8). The procedure was performed in duplicate.

### 3.13. Antibacterial Capacity of Edible Films

The antibacterial capacity of the edible films was evaluated with the diffusion disk method previously described in Section 3.9.

### 3.14. Statistical Analysis

Average and standard deviation were calculated for the different tests. Additionally, an ANOVA (Tukey’s multiple comparison test at 95% confidence level) was performed to establish whether there were significant differences between the means of values obtained from the different analyses. Pearson’s correlation tests were performed between the variables of this study. In all cases, Statgraphics Centurion XVIII software was employed.

## 4. Conclusions

In this study, for the first time, extracts rich in anthocyanins obtained from *L. chequen* fruit by ultrasound-assisted NADES extraction were incorporated into edible films. It was observed that extracts with higher anthocyanin contents that were made with glycerol-based NADES allowed us to create edible films, indicating that such materials can improve the antioxidant and antibacterial properties of packaging materials.

The antibacterial activity of the extracts indicated that most of them exhibited an effect against Gram-negative (−) and Gram-positive (+) strains. The antioxidant capacity indicates that these extracts have similar potential to extracts of other berries which may be related to the fact that organic acid-, sugar-, and glycerol-based NADES showed improved extraction of anthocyanins, mainly derivatives of delphinidins, cyanidins, and petunidins, when compared to ethanolic extracts.

These results are crucial to reveal the potential of native Chilean flora, as well as the development of new research, regarding the properties of these fruits. Possibilities are opened up to research and develop new products from these extracts as types of additives for active films, since they have antibacterial and antioxidant properties which allow the creation of active and sustainable packaging with the potential to be used in the food industry.

## Figures and Tables

**Table 1:** Anthocyanin content in *L. chequen* (Molina) A. Gray fruit using natural deep eutectic solvents (NADES) of different compositions.

NADES (Code)	Monomeric Anthocyanins (mg/g dw)	Total Anthocyanins (mg/g dw)	Total Anthocyanins (mg Eq Cyanidin 3-Glucoside/100 g dw)
La-Gly 1:1	1.16 ± 0.02 ^e,f^	1.28 ± 0.02 ^c^	127.7 ± 2.1 ^c^
La-Gly 1:2	2.22 ± 0.04 ^b,c,d^	2.40 ± 0.04 ^a,b,c^	240.4 ± 4.2 ^a,b,c^
La-Gly 2:1	2.07 ± 0.04 ^c,d^	2.16 ± 0.51 ^b,c^	216.4 ± 5.1 ^b,c^
Ta-Gly 1:2	0.66 ± 0.00 ^f^	0.81 ± 0.04 ^d^	81.1 ± 4.2 ^e^
Ta-Gly 1:3	1.65 ± 0.04 ^d,e^	1.76 ± 0.02 ^c,d^	175.8 ± 2.1 ^c,d^
Ta-Gly 1:4	2.90 ± 0.02 ^a,b^	3.05 ± 0.10 ^a,b^	305.0 ± 10.6 ^a,b^
La-Glu 8:1	2.76 ± 0.42 ^a,b,c^	3.30 ± 0.04 ^a^	330.6 ± 4.2 ^a^
Gly-Glu 8:1	2.94 ± 0.08 ^a^	3.06 ± 0.08 ^a,b^	306.5 ± 8.5 ^a,b^
ClC-Ca 5:4	2.07 ± 0.08 ^c,d^	2.27 ± 0.08 ^a,b,c^	226.9 ± 2.0 ^a,b,c^
ClC-Gly 4:6	3.13 ± 0.35 ^a^	3.28 ± 0.46 ^a,b^	327.6 ± 46.7 ^a,b^
Ethanol	0.98 ± 0.06 ^g^	1.16 ± 0.03 ^d^	116.2 ± 3.6 ^d^

La, lactic acid; Gly, glycerol; Ta, tartaric acid; Glu, glucose; ClC, choline chloride; Ca, citric acid. Different letters in columns indicate significant difference (Tukey’s honestly significant difference (HSD), *p* < 0.05).

**Table 2:** Extracts with higher anthocyanin content (polymeric, density, and CIELAB parameters of color).

Solvents	Polymeric Color	Color Density	% Polymeric Color	L*	a*	b*	C*	*H°*
La-Gly 1:2	0.5 ± 0.1 ^c^	2.3 ± 0.4 ^a^	25.5 ± 0.0 ^e^	19.4 ± 2.0 ^c^	19.7 ± 3.7 ^a^	7.3 ± 0.2 ^b,c^	21.0 ± 3.4 ^a^	20.6 ± 4.2 ^b^
Ta-Gly 1:4	1.0 ± 0.1 ^b^	3.6 ± 1.4 ^a^	28.4 ± 0.1 ^c^	18.8 ± 0.1 ^c^	18.1 ± 0.4 ^a^	7.9 ± 4.2 ^a,b^	19.9 ± 2.1 ^a,b^	22.9 ± 10.6 ^b^
La-Glu 8:1	1.4 ± 0.4 ^a^	4.9 ± 0.9 ^a^	29.4 ± 0.0 ^c^	21.5 ± 0.3 ^c^	21.1 ± 1.4 ^a^	5.6 ± 2.5 ^b,c^	20.4 ± 1.5 ^a^	15.9 ± 6.2 ^c^
Gly-Glu 8:1	1.7 ± 1.5 ^a^	3.0 ± 1.5 ^a^	55.2 ± 0.2 ^a^	23.9 ± 1.9 ^b^	8.9 ± 0.1 ^c^	2.1 ± 1.6 ^c^	9.2 ± 0.4 ^c^	13.3 ± 9.8 ^c^
ClC-Ca 5:4	0.7 ± 0.2 ^c^	2.4 ± 1.0 ^a^	31.1 ± 0.1 ^c^	19.4 ± 0.3 ^c^	22.8 ± 0.0 ^a^	10.5 ± 2.9 ^a^	25.2 ± 1.2 ^a^	24.6 ± 5.8 ^b^
ClC-Gly 4:6	0.8 ± 0.3 ^c^	1.9 ± 1.2 ^a^	40.0 ± 0.1 ^b^	27.5 ± 0.5 ^a^	7.1 ± 0.6 ^c^	8.9 ± 3.4 ^a,b^	11.5 ± 3.0 ^c^	50.6 ± 8.1 ^a^
Ethanol	0.6 ± 0.1 ^c^	2.2±0.9 ^a^	27.2 ± 0.1 ^d^	26.6± 0.3 ^a^	13.9 ± 0.1 ^b^	13.8 ± 1.5 ^a^	19.6 ± 1.6 ^a,b^	44.5 ± 5.1 ^a^

Different letters in columns indicate significant differences (Tukey’s HSD, *p* < 0.05). L*, luminosity; a*, reddish and green tones; b*, blue and yellowish tones; C*, chromaticity; *H°*, hue angle. All parameters are dimensionless.

**Table 3:** Effect of anthocyanins obtained with different NADES on antioxidant capacity and bacterial inhibition diameter of edible film.

	Antioxidant Capacity		Antibacterial Capacity		
Edible Films	DPPH Inhibition (%)	ABTS Inhibition (%)	*E. coli* (mm)	*S. typhi* (mm)	*S. aureus* (mm)
Control (C)	7.35 ± 2.04 ^a^	21.72 ± 0.74 ^b^	ND	ND	ND
C + (La-Gly 1:2)	7.38 ± 2.61 ^a^	30.50 ± 1.95 ^a^	5.0 ±0.1 ^b^	ND	ND
C + (Ta-Gly 1:4)	8.71 ± 4.65 ^a^	22.47 ± 0.00 ^b^	5.0 ± 0.1 ^b^	ND	6.0 ±0.1 ^b^
C + (La-Glu 8:1)	10.81±1.99 ^a^	21.73 ± 2.18 ^b^	6.0 ± 0.1 ^a^	6.0 ± 0.1 ^a^	6.0 ± 0.1 ^b^
C + (Gly-Glu 8:1)	7.44 ± 2.52 ^a^	23.85 ± 2.11 ^a,b^	5.0 ± 0.1 ^b^	ND	6.0 ± 0.2 ^b^
C + (ClC-Gly 4:6)	7.26 ± 2.77 ^a^	20.87 ± 2.11 ^b^	5.0 ± 0.1 ^b^	ND	7.0 ± 0.2 ^a^

Different letters in columns indicate significant difference (Tukey’s HSD, *p* < 0.05). ND, no inhibition diameter.

## Supplementary Materials

The following are available online, Figure S1: Chromatogram of ethanolic extract of *L. chequen* obtained by HPLC–DAD, Figure S2: Chromatogram of extract ClC-Gly 4:6 of *L. chequen* obtained by HPLC–DAD, Figure S3: Chromatogram of extract Gly-Glu 8:1 of *L. chequen* obtained by HPLC–DAD, Table S1: Details of NADES components, Table S2: Conditions of HPLC-DAD analysis for the identification of anthocyanins, Table S3: Antioxidant and antibacterial indices of extracts analyzed.

## Sample Availability

Samples of the compounds are available from the authors.
