# Supplementary material for: Ultrasound-Assisted Extraction of Anthocyanins Using Natural Deep Eutectic Solvents and Their Incorporation in Edible Films
Source: Molecules. 2021 Feb 12;26(4):984. doi: 10.3390/molecules26040984 (PMC7918079; doi:10.3390/molecules26040984)
Supplement: Supplementary file 1 [file molecules-26-00984-s001.pdf]

Supplemental Material

ULTRASOUND ASSISTED EXTRACTION OF ANTHOCYANINS USING NATURAL DEEP EUTECTIC SOLVENTS AND THEIR INCORPORATION IN EDIBLE FILMS

Patricia Velásquez [1,2], Daniela Bustos [1], Gloria Montenegro [2] and Ady Giordano [1,*]

[1] Departamento de Química Inorgánica, Facultad de Química y de Farmacia; dcbustos@uc.cl (D.B.); agiordano@uc.cl (A.G.); pdvelasquez@uc.cl (P.V.)

[2] Departamento de Ciencias Vegetales, Facultad de Agronomía e Ingeniería Forestal; gmonten@uc.cl (G.M.); pdvelasquez@uc.cl (P.V.)

* Correspondence: agiordano@uc.cl (A.G); Tel.: (+56-2) 2354 1542 (A.G.)

Received: date; Accepted: date; Published: date

Extracts rich in bioactive compounds added to edible films have allowed the development of active packaging that increases the shelf life of food. However, it is necessary to search for solvents that are nontoxic and not harmful to the environment, with natural deep eutectic solvents (NADES) being an attractive and easily synthesized alternative. This research aimed to design NADES by lyophilization to be used in the extraction of anthocyanins from the Chilean *Luma chequen* (Molina) A. Gray berry, and subsequently add them to the matrix of edible ß-carrageenan films. For this purpose, ultrasound-assisted extraction (UAE) was used and the anthocyanin content was evaluated with the pH differential method. The antioxidant capacity of extracts was determined by DPPH assay and the antibacterial capacity by diffusion agar tests. The results obtained indicate that the designed NADES are efficient at extracting anthocyanins, reaching concentrations between 81.1 and 327.6 mg eq cyanidin 3-glucoside/100 g dw of *L. chequen* (Molina) A. Gray. The extracts reached inhibition diameters between 5 and 34 mm against *E. coli*, *S. aureus*, and *S. typhi* strains. Once the extracts were incorporated into ß-carrageenan films, active edible films with antioxidant and antibacterial capacities were obtained.

Keywords: carrageenan; berries; green chemistry; green extraction; active compounds; medicinal plants; active packaging

.

Table S1: NADES composition and properties.

NADES (codes)	Components	Molar relation	%H2O	pH	Density
La-Gly 1:1	Lactic acid-Glycerol	1:1	10	0,44	1,20
La-Gly 1:2	Lactic acid-Glycerol	1:2	20	0,66	1,16
La-Gly 2:1	Lactic acid-Glycerol	2:1	30	0,27	1,09
Ta-Gly 1:2	Tartaric acid- Glycerol	1:2	26	0,51	1,53
Ta-Gly 1:3	Tartaric acid- Glycerol	1:3	36	0,5	1,26
Ta-Gly 1:4	Tartaric acid- Glycerol	1:4	46	0,52	1,27
La-Glu 8:1	Lactic acid -Glucose	8:1	40	0,81	1,16
Gly-Glu 8:1	Glycerol -Glucose	8:1	40	4,45	1,12
*ClC-Ca 5:4	Choline chloride – Citric acid	5:4	10	1,00	1,26
*ClC-Gly 4:6	Choline chloride - Glycerol	4:6	02	6,10	1,19

*Commercial products

Table S2: Conditions of analysis HPLC-DAD for the anthocyanin's analysis

Time (min)	Formic acid 0,1 (%)	Methanol (%)
0	90	10
4	90	10
4-25	75	25
25-40	90	10

Table S3: Antioxidant and antibacterial capacities of *L. chequen* (Molina) A. Gray extracts analyzed

Extracts	IC_{50} (mg /mL)	MIC *E. coli* (µg/mL)	MIC *S. typhi* (µg/mL)	MIC *S. aureus* (µg/mL)
(La-Gly 1:2)	3,45	0,878	0,439	1,75
(Ta-Gly 1:4)	4,21	0,878	0,439	0,439
(La-Glu 8:1)	5,56	0,878	0,439	0,439
(Gly-Glu 8:1)	5,53	-	-	-
(ClC-Ca 5:4)	6,32	0,439	0,439	0,439
(ClC-Gly 4:6)	5,11	-	-	-

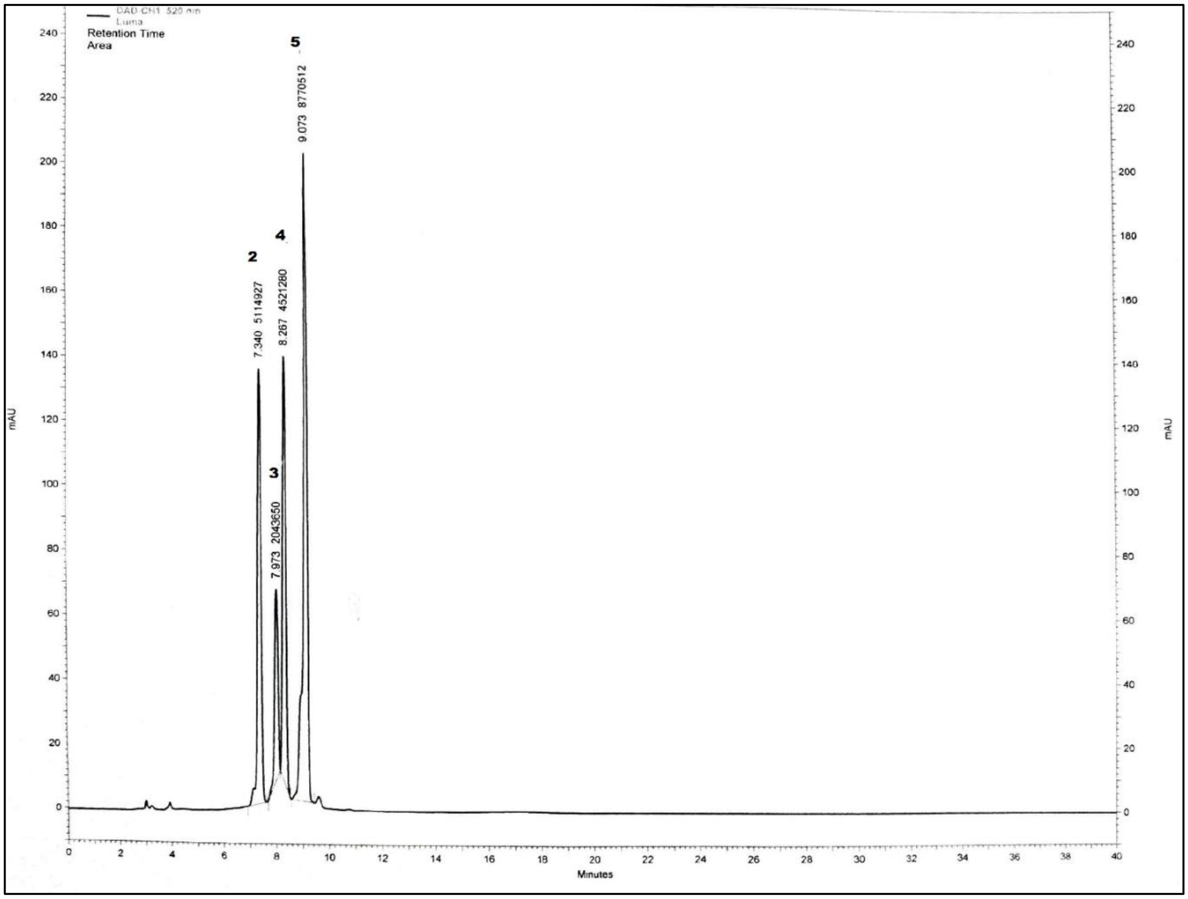

Figure S1: Chromatogram of ethanolic extract of *L. chequen* (Molina) A. Gray obtained by HPLC−DAD

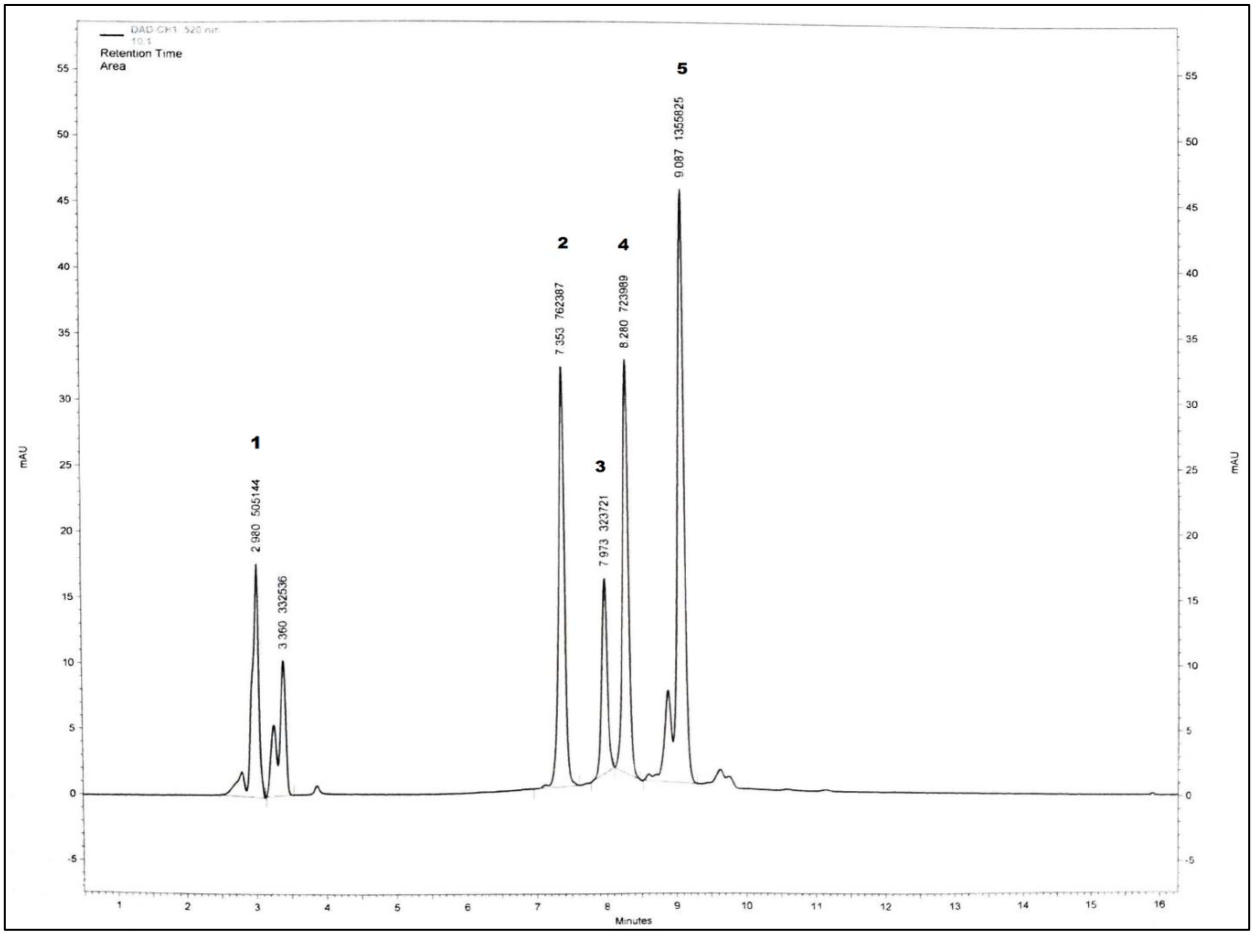

Figure S2: Chromatogram of extract ClC-Gly 4:6 of *L. chequen* (Molina) A. Gray obtained by HPLC–DAD.

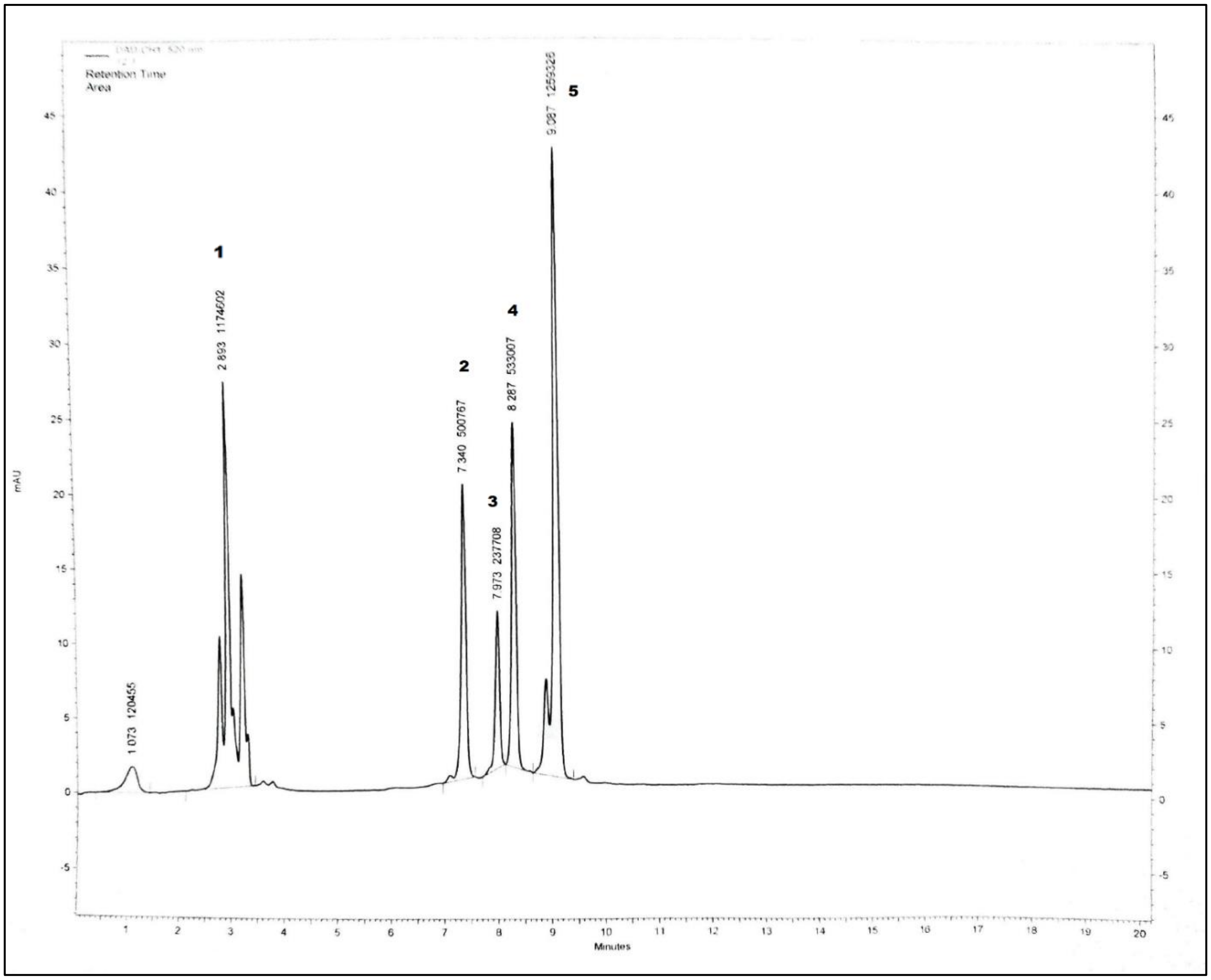

Figure S3: Chromatogram of extract Gly-Glu 8:1 of *L. chequen* (Molina) A. Gray obtained by HPLC−DAD.
